# pH-cycling models for *in vitro* evaluation of the efficacy of fluoridated dentifrices for caries control: strengths and limitations

**DOI:** 10.1590/S1678-77572010000400002

**Published:** 2010

**Authors:** Marília Afonso Rabelo BUZALAF, Angélica Reis HANNAS, Ana Carolina MAGALHÃES, Daniela RIOS, Heitor Marques HONÓRIO, Alberto Carlos Botazzo DELBEM

**Affiliations:** 1 DDS, MS, PhD, Full Professor, Department of Biological Sciences, Bauru School of Dentistry, University of São Paulo, Bauru, SP, Brazil.; 2 DDS, MS, PhD, Department of Biological Sciences, Bauru School of Dentistry, University of São Paulo, Bauru, SP, Brazil.; 3 DDS, MS, PhD, Assistant Professor, Department of Biological Sciences, Bauru School of Dentistry, University of São Paulo, Bauru, SP, Brazil.; 4 DDS, MS, PhD, Assistant Professor, Department of Pediatric Dentistry, Orthodontics and Community Health, Bauru School of Dentistry, University of São Paulo, Bauru, SP, Brazil.; 5 DDS, MS, PhD, Associate Professor, Department of Pediatric Dentistry, Alfenas Federal University, Alfenas, MG, Brazil.; 6 DDS, MS, PhD, Associate Professor, Department of Pediatric Dentistry, Araçatuba Dental School, Paulista State University, Araçatuba, SP, Brazil.

**Keywords:** Dental caries, Dentifrices, Efficacy, Fluorides, In vitro

## Abstract

Despite a plethora of *in situ* studies and clinical trials evaluating the efficacy of fluoridated dentifrices on caries control, *in vitro* pH cycling models are still broadly used because they mimic the dynamics of mineral loss and gain involved in caries formation. This paper critically reviews the current literature on existing pH-cycling models for the *in vitro* evaluation of the efficacy of fluoridated dentifrices for caries control, focusing on their strengths and limitations. A search was undertaken in the MEDLINE electronic journal database using the keywords "pH-cycling", "demineralization", "remineralization", "*in vitro*", "fluoride", "dentifrice". The primary outcome was the decrease of demineralization or the increase of remineralization as measured by different methods (e.g.: transverse microradiography) or tooth fluoride uptake. Inclusion of studies, data extraction and quality assessment were undertaken independently and in duplicate by two members of the review team. Disagreements were solved by discussion and consensus or by a third party. One hundred and sixteen studies were included, of which 42 addressed specifically the comparison of dentifrices using different pH-cycling models. The other studies included meta-analysis or reviews, data about the effect of different fluoride sources on de-remineralization, different methods for analysis de-remineralization and chemical variables and characteristics of dental hard tissues that might have influence on de-remineralization processes. Generally, the studies presented ability to detect known results established by clinical trials, to demonstrate dose-related responses in the fluoride content of the dentifrices, and to provide repeatability and reproducibility between tests. In order to accomplish these features satisfactorily, it is mandatory to take into account the type of substrate and baseline artificial lesion, as well as the adequate response variables and statistical approaches to be used. This critical review of literature showed that the currently available pH-cycling models are appropriate to detect dose-response and pH-response of fluoride dentifrices, and to evaluate the impact of new active principles on the effect of fluoridated dentifrices, as well as their association with other anti-caries treatments.

## INTRODUCTION

A study model is a process that simulates some real-world phenomenon of interest, thus allowing the researcher to derive information about this phenomenon^73^. Despite a plethora of *in situ* studies and clinical trials, *in vitro* models are the most commonly employed methods in Cariology research. Among *in vitro* protocols, pH-cycling models involve exposure of dental substrates (enamel or dentin) to combinations of demineralization and remineralization. These combination experiments are designed to mimic the dynamics of mineral loss and gain involved in caries formation^106^, which is an important advantage of pH-cycling models. Other advantages include the high level of scientific control and the resulting lower variability intrinsic to *in vitro* models, as well as the smaller sample size required. Additionally, the response variables that can be employed in pH-cycling models are more sensitive than those available for use in the clinical situation. Due to these key advantages, pH-cycling models have helped improving the understanding of the caries process and the possible mechanisms by which fluoride exerts its anti-caries effect. Furthermore, they are broadly used in profile studies for rapid and inexpensive testing of developing and recently marketed products^106,116^. The role of pH-cycling models is therefore to facilitate the generation of sufficient quantitative data to give investigators the confidence to appropriately design clinical trials^18^.

However, pH-cycling models as all *in vitro* protocols have important limitations: (1) they are unable to completely simulate the complex intraoral conditions leading to caries development, even when "artificial mouth" systems, bacterial biofilms and saliva are employed. This is particularly relevant for testing fluoridated dentifrices with monofluorophosphate (MFP), since the enzyme systems required for MFP hydrolysis are present in saliva and plaque *in vivo*, but are absent in most *in vitro* test methods; (2) they cannot mimic solid surface area/solution ratios or the saliva/plaque fluid composition encountered *in vivo*^107^, since different oral surfaces are bathed in different volumes and source combinations of saliva, (3) there are artifacts associated with the choice of substrate and test conditions, particularly the time periods of de- and remineralization, which are much faster than those expected to occur in *in vivo* conditions^106^; and (4) they are not able to adequately simulate topical use and clearance of products from the oral cavity. While dentifrices are typically slurried to simulate dilution during brushing, the uptake and reactivity of fluoride are consistently lower *in vivo* than *in vitro*, which may result in inaccurate assessments of the anti-caries potential of formulations directed toward enhancement of fluoride delivery^107^. All these limitations must be kept in mind when data from pH-cycling studies are intended to be extrapolated for the clinical situations.

This paper critically reviews the current literature on existing pH-cycling models for the *in vitro* evaluation of the efficacy of fluoridated dentifrices for caries control, focusing on their strengths and limitations. Additionally, the impact of the characteristics of previous artificial caries lesions on subsequent de-remineralization, the response variables usually chosen, and the American Dental Association (ADA) guidelines^1,2^ for this kind of tests are considered. Finally, studies involving different pH-cycling protocols are compared, considering separately those focusing on fluoride dose-response or pH-response of the dentifrices, type of fluoride compound present in the dentifrice, the impact of new active principles on the effect of fluoridated dentifrices, as well as their association with other anti-caries treatments. The validity of the pH-cycling protocols is discussed in the light of data from clinical studies, whenever possible. Based on the currently available literature, future perspectives in pH-cycling protocols are also included in the discussion.

## METHODS

A search was undertaken in the MeDLINe electronic journal database using the keywords "pHcycling", "demineralization", "remineralization", "*in vitro*", "fluoride", "dentifrice". The primary outcome was the decrease of demineralization or the increase of remineralization as measured by different methods (e.g.: microhardness, microradiography, polarized light microscopy) or tooth fluoride uptake.

One hundred and sixteen papers referring to the following issues were retrieved: 1) the comparison of dentifrices using different pH-cycling models; 2) meta-analysis/reviews about methods or the effect of fluoride on dental de-remineralization; 3) the effect of different fluoride sources on deremineralization *in vitro*; 4) different methods to analysis de-remineralization and; 5) chemical variables and characteristics of dental hard tissues that might have influence on de-remineralization process.

Inclusion of studies, data extraction and quality assessment were undertaken independently and in duplicate by two members of the review team. Disagreements were resolved by discussion and consensus or by a third party.

## RESULTS AND LITERATURE REVIEW

The genesis of modern pH-cycling models was produced by ten Cate and Duijsters^87^ (1982). In typical pH-cycling studies for testing the efficacy of fluoridated dentifrices, a dental substrate (enamel or dentin, from permanent or primary teeth, from human or bovine origin) is sequentially exposed to demineralizing and remineralizing solutions with intermediary treatments with the dentifrices.

**Figure 1:**
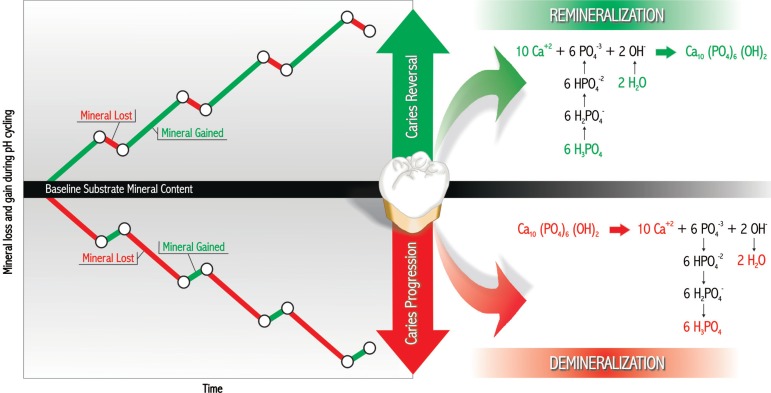
Classification of *in vitro* pH-cycling models according to the flux of minerals. Modified from White^106^ (1995)

*In vitro* pH-cycling models can generally be classified into progression (demineralizing) or reversal (remineralizing) models depending on the flux of mineral from or to the dental substrate, respectively^106^, as shown in Figure 1. The demineralizing models usually employ an initially sound substrate and the response variable will analyze the potential of the dentifrice to reduce the loss of mineral from the substrate to the demineralizing solution or the gain of mineral from the remineralizing solution. Demineralizing models can also employ substrates with artificial caries lesion to measure the extent of further demineralization. In remineralizing models, dental substrates with artificial caries lesions are used and the response variables will measure the mineral gain in the lesions as a consequence of the treatment with the dentifrices.

Some important methodological aspects have been found in the literature review. They will be presented and discussed below in order to make easier the choice for appropriate protocols for the incoming studies as well as the adequate interpretation of the results of existing publications by the readers. Finally, the data from studies about the effect of fluoride dentifrices on deremineralization will be presented in tables and discussed in the text. The future perspectives for studies using pH-cycling as an *in vitro* model will also be addressed.

### Type of dental substrates that can be used in ph-cycling models

Excellent reviews to guide the choice of dental substrates for *in vitro* and *in situ* studies have been presented by Mellberg^60^ (1992) and Ogaard and Rolla^68^ (1992). Regarding the origin of the substrates, human teeth can be regarded as the most appropriate source from the perspective of clinical relevance. However, their composition is variable, due to genetic influences, environmental conditions (diet, fluoride exposure, previous caries challenge) and age (post-eruptive maturation and dentin sclerosis). These differences lead to large variations in their response under acidic challenges. Among the different types of human teeth, permanent molars and premolars are the most often employed teeth. Primary teeth are only occasionally used because it is difficult to obtain them and they have a small surface for experimental manipulation. When primary teeth are used, it must be taken into account that the progression of lesions *in vitro* is faster than would occur for permanent teeth^114^. Regarding the age, impacted or partially erupted teeth are more porous than teeth that have been exposed to the oral cavity for a longer time and caries progression should be expected to occur in a faster rate^10^. In addition, dentin sclerosis, which occurs with age, can alter the dentin susceptibility to caries development since a gradual mineralization of the peritubular dentin occurs, eventually resulting in complete obliteration of the tubules^79^.

On the other hand, bovine teeth are more readily available and have a more uniform composition when compared to human teeth, thus providing a less variable response to both cariogenic challenge and anti-caries treatments, such as fluoridated dentifrices^60^. Additionally, bovine teeth have a bigger surface area which makes easier experimental manipulation. Furthermore, bovine teeth present higher porosity, which allows a faster diffusion of ions to the demineralized area ^26,28,55^. Thus, longer experimental periods might compromise the ability of the model to show fluoride dose-response. Although bovine enamel is more porous than human enamel, which leads to faster demineralization and remineralization, these differences result in quantitative and not qualitative differences in behavior. Additionally, the artificial caries lesions produced from bovine teeth have a mineral distribution and structure that resembles lesions produced from human teeth, both for enamel and dentin^20,28,37,60^. Thus, bovine tooth can be considered an acceptable alternative to human tooth in Cariology research and may offer advantages to human tooth by decreasing the response time and variability of the hard tissue substrate in the model^60^.

Considering the different types of mineralized dental tissues, enamel and dentin have very different structures and compositions, which interfere in their susceptibilities to dental caries. Basically, permanent enamel is composed by mineral (85% volume) in the form of hydroxy- or fluorapatite crystals organized in prisms. Upon a cariogenic challenge (4.5<pH<5.5), hydroxyapatite crystals are dissolved from the subsurface, while fluorapatite crystals are deposited at the surface, originating a subsurface lesion. The dissolution process is merely a chemical event.

Permanent dentin, however, contains 47% apatite, 33% organic components (90% collagen and 10% non-collagenous proteins) and 20% water by volume. The mineral phase is hydroxyapatite, similar to enamel, but the crystallites have much smaller dimensions. The hexagonal dentinal crystallites are 3-30 nm in cross-section and about 50 nm in length. This results in a much larger surface area to crystallite volume ratio and therefore a more reactive mineral phase. The organic matrix is mainly composed of collagen. It is present as a very structured triple helix of three intertwined polypeptide chains. In addition, there are many non-collagenous (phosphoproteins, phospholipids and proteoglycans) components that determine the matrix properties. These compounds play a role in the nucleation and regulation of mineral formation during odontogenesis^90^. Then they may interfere with demineralization and remineralization processes by a similar mechanism of action. There is a synergism between matrix and apatite. The mineral phase can only be partially dissolved during an acid attack, while the matrix cannot be digested by enzymatic action while its surface is protected by apatite. Dentin caries is therefore a biochemical process characterized initially by the dissolution of the mineral part thus exposing the organic matrix to breakdown^50,66,79^ by bacteria-derived enzymes and host-derived enzymes, such as the matrix metalloproteinases (MMPs) present in dentin and saliva^15,100^. The dentin demineralization rate decreases when the amount of degradable collagen increases, whereby the demineralized matrix is attributed to hamper ionic diffusion into and out of the demineralizing area^49,50^. However, it should be remembered that during caries lesion formation *in vivo,* teeth with a vital pulpo-dentinal organ will respond to most exogenous stimuli through the apposition of minerals along and within the dentinal tubules^32^. This phenomenon, together with the outward flow of dentinal fluid from the pulp, may be expected to significantly reduce the rate of *in vivo* lesion progression in dentin compared to *in vitro* situation^80^.

**Figure 2:**
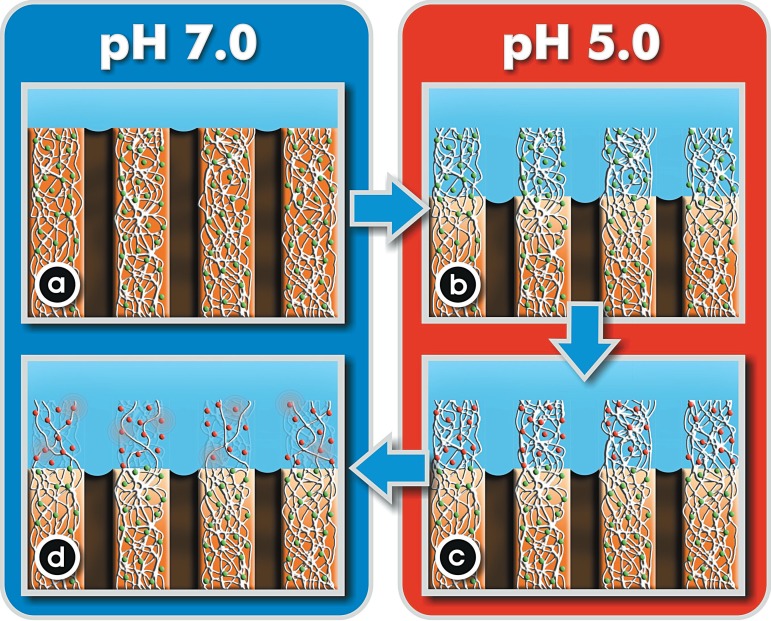
Schematic illustration of the process of dentin dissolution by bacterial acids. Under resting conditions (pH 7.0) both the mineral and organic matrix (containing inactive MMPs, green dots) are intact (a). Upon a cariogenic challenge (pH 5.0), the apatite is dissolved thus exposing the organic matrix to degradation by salivary and dentin MMPs (green dots represent MMPs) (b). The low pH also activates MMPs, which are represented as red dots (c). The subsequent neutralization by salivary buffer systems enhances the degrading activity of the organic matrix by MMPs (d)^100^

It has been shown that MMPs get activated when the pH drops in the presence of acids from cariogenic challenges. The subsequent neutralization by salivary buffer systems enhances the degrading activity of the organic matrix^100^ (Figure 2). It is noteworthy that dentin matrix degradation occurs when bacterial collagenase is added to remineralizing solution but not to demineralizing solution in pH-cycling models^47^, i.e. the activity of bacterial collagenase added to demineralization solution is lost, while the activity of host MMPs is enhanced at low pH, emphasizing the importance of host collagenases^100^. Thus, pH-cycling models without the addition of collagenases or gelatinases can only simulate the chemical events involved in root dentin caries, since they do not take into consideration the biochemical role of salivary MMPs in the degradation of the demineralized organic matrix. Additionally, when simulating dentin caries *in vitro*, it is also important to use freshly extracted teeth and store these teeth properly (0.02% NaN_3_, 0.9% NaCl solution, at 4ºC)^70^ in order to assure that the activity of dentin MMPs is preserved.

In addition to the necessity of degradation of the organic matrix for dentin caries progression, dentin de- and remineralization has many other characteristics that differ from enamel de- and remineralization: (1) dentin is more susceptible to caries attack than is enamel, with a critical pH more than one pH-unit higher than that for enamel^41^; (2) dentin demineralizes faster and remineralizes slower than enamel under the same experimental conditions^4,86^; (3) more concentrated fluoride is needed for remineralization of dentin than for enamel^38^; (4) dentin contact area with cariogenic acids is bigger than that of enamel and hence dentin is apparently much more permeable to acids, with demineralization taking place at a relatively large depth, while mineral deposition is restricted to the outer layers. If the crystallites surrounding the diffusion channels (tubules) are coated with a fluoride-rich mineral (due to the use of fluoride dentifrice), the acids will bypass these relatively resistant minerals, while mineral and fluoride ions will readily be deposited. Thus, the lesion front in dentin moves deeper, while the surface layer becomes broader. In enamel, on the other hand, diffusion is much slower and allows acids to "sidestep" into smaller intraprismatic porosities and dissolve crystallites that are still unaffected by either acid or fluoride. Thus, mineral uptake and loss occur at similar depths for enamel lesions, while for dentin lesions mineral uptake is predominant at the surface and mineral loss at the lesion front^86^.

It must be also pointed out that the dental substrates have usually to be polished before the beginning of the experiment in order to produce more uniform and homogeneous surfaces that can be more accurately standardized, resulting in abraded surfaces. This procedure is essential for some response variables such as surface hardness analysis. The removal of the outermost fluoride-rich enamel layer will render a faster demineralization of the subsurface during the subsequent pH-cycles^5,96^.

### Characteristics of Artificial Caries Lesions that May Affect De- and Remineralization in ph-Cycling Models

**Figure 7:**
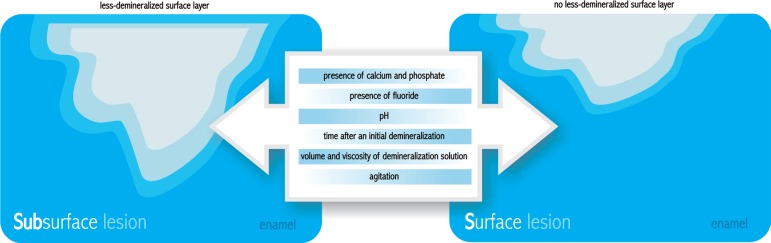
Factors that affect the formation of subsurface lesions (caries-like) and surface-softened lesions (erosion-like)

In some studies, artificial caries lesions are initially produced by immersion of the substrates in buffered lactate or acetate gels^110^ or solutions, undersaturated in respect to apatite, with a pH ranging between 4.4 and 5.0, for a time ranging between 16 h and 28 days (Figures 3, 4, 5 and 6). These distinct protocols will lead to different types of lesions formed (surface softened lesions, also known as erosion-like lesions, or subsurface lesions, also called caries-like lesions) (Figure 7). The distinct integrated mineral loss (∆Z) and depth at baseline^54-56^ in these lesions can have a profound impact on the subsequent de-and remineralization rates thus affecting the performance of the tested products.

**Figura 3:** pH-cycling studies evaluating the dose-response or pH-response of fluoridated dentifrices for caries prevention

**PROTOCOL**	**DENTAL TISSUE**	**RESPONSE VARIABLE**	**RESULTS**
pH-cycling	Human enamel	CSH x TMR [Ten Cate, et al.^92^ (1985)]	The NaF dentifrice was found to be extremely effective in reducing the progression of caries in enamel
Re:14d, 37°C, on the weekends and before De			
De: for 6h/day in 40 ml of acid buffer containing 2.0 mM Ca, 2.0 mM PO_4_, 0.075 M acetate, pH 4.3			
Treatment: with slurry 1:4 in water for 5 min/Re for 17 h in 20 mL of a mineralizing solution containing 1.5 mM Ca, 0.9 mM PO_4_, 0.15 M KCl and 20 mM cacodylate buffer, pH 7.0 [as described by Featherstone, et al.^29^ (1986)]			
Ref: White and Featherstone^113^ (1987)			
Method 1	Human sound and bovine carious enamel	Method 1:	F dentifrice is very effective in inhibiting lesion formation in initially sound enamel as well as in inhibiting lesion progression .
pH cycling sound human enamel:		CSH	The effect of F dentifrice on prevention of demineralization and increase of remineralization depends on the type of lesion
De: 6h/day (75mM acetic acid, 2mM Ca(NO_3_)_2_, 2mM KH_2_PO_4_, pH 4.3, 20 mL/sample)		Method 2:	
Re: 17 h/day (20 mM cacodylate buffer at pH 7.0, 130 mM KCl, 1.5 mM Ca(NO_3_)_2_, 0.9 mM KH_2_PO_4_, 20 mL/ sample). Total:15 d (remineralizing solution, 37ºC, on the weekend)		F analysis after acid etch biopsy (samples) and Ca loss and uptake (solutions) by AAS	
F treatment: slurry 1:3 water, 5 mL/ 5 min, under agitation before or after De.			
			
Artificial caries before Method 2			
(7 d, 10 mL, 37ºC): calcium-phosphate-fluoride-acetate system (2.2 mM Ca(NO_3_)_2_, 2.2 mM KH_2_PO_4_, 0.5 mM F, 50 mM acetate, pH 4.5) or 0.2 mM MHDP in 100 mM lactate buffer (pH 4.5)			
			
Method 2			
pH cycling carious bovine enamel:			
De: 4 weeks -3 h/day (50 mM acetic acid, 1.5 mM Ca(NO_3_)_2_, 0.9 mM KH_2_PO_4_, pH 4.5-4.75)			
Re: 21 h/day (20 mM cacodylate buffer at pH 7.0, 130 mM KCl, 1.5 mM Ca(NO_3_)_2_, 0.9 mM KH_2_PO_4_, 20 mL/ sample)			
F treatment: as described above			
Ref: Ten Cate, et al.^93^ (1988)			
Test 1: 5 min test solution (2 mL), 1 min water (2 mL)	Bovine enamel with salivary pellicle	Calcium analysis by AAS (De-Re solutions)	Residual salivary [F] by water fluoridation or toothpaste may give some protection to enamel demineralization
De: 1 h acid treatment (50 mM acetic acid, 1.5 mM KH_2_PO_4_, pH 5)	(5-10mm^2^)		
Re: 1 h remineralization (20 mM cacodylic acid, 1.5 mM KH_2_PO_4_, pH 7).			
Total: 8 cycles (18 h)			
Test 2: Re solution (1 h/overnight), water rinse (1 min) and acid solution (1 h) during 3 days			
Ref: Page^69^ (1991)			
Artificial caries: 8% methylcelulose gel, 0.1 M lactic acid (pH 4.6/7 d -Enamel and pH4.8/5 d-dentin)	Bovine	Calcium uptake and loss by AAS (De and Re solutions)/ TMR and loosely and firmly bound F (samples)	Low F levels - less effective to inhibit caries lesion in dentin than in enamel
pH cycling:	enamel and		F dentifrice has a more pronounced effect on dentin than on enamel
De:(3 mL, 1.5 mM CaCl_2_, 0.9 mM KH_2_PO_4_and 50 mM acetic acid, pH 5.0, 6x0.5 h/day)^2^	dentin		
Re: (3 mL, 1.5 mM CaCl2, 0.9 mM KH_2_PO_4_, 130 mM KCl and 20 mM Hepes, pH 7.0, 6x 2.5 h/day, overnight and weekend)	(22 mm^2^)		
3 days without treatment/ 7 days with treatment			
Treatment: dentifrice slurry (1:3 in water, 5 min) x 3 |jM F in de-remineralizing solutions x deionized water (5 min)			
Ref: Ten Cate, et al.^86^ (1995)			
Caries lesion: 96 h De solution	Primary enamel		The 10-day pH cycling model is inappropriate for primary teeth de/remineralization analysis.
pH-cycling:	(1-mm)		Positive regarding the treatment
De: 2.2 mM CaCl_2_, 2.2 mM NaH_2_PO_4_, 0.05 M acetic acid, pH 4.4 . 2x3 h/day			
Re:1.5 mM CaCl_2_, 0.9 mM NaH_2_PO_4_, 0.15 M KCl ,pH 7.0, 2 h between De, according to Ten Cate and Duijsters^87^ (1982)			
Treatment: slurry (30 mL deionized water) for 1min before 1^st^ De and before and after 2 ^nd^De		TMR and PLM	
Model I: 10-day pH-cycling			
Model II: 7-day pH-cycling			
Ref: Thaveesangpanich, et al.^95^ (2005)			
Caries lesion: 96 h De solution	Primary enamel		Positive results regarding the treatment.
pH-cycling:	(1-mm window)		Both 10-day (containing 0.25ppm F) and 7-day (without F) pH-cycling models were suitable for studying caries lesion progression in primary teeth
De: 2.2 mM CaCl_2_, 2.2 mM NaH_2_PO_4_, 0.05M acetic acid, pH 4.4 . 2x3 h/day		TMR and PLM	
Re:1.5 mM CaCl_2_, 0.9 mM NaH_2_PO_4_, 0.15 M KCl, pH 7.0, 2 h between DE, according to Ten Cate and Duijsters^87^ (1982)			
Treatment: slurry (30 ml deionized water) for 1 min before 1 ^st^De and before and after 2 ^nd^De			
Model I: as mentioned above, 7-day pH-cycling			
Model II: 0.25 ppm F added to de- and re- solutions, pH of de solution adjusted to 4.5, 10-day pH-cycling			
Ref: Thaveesangpanich, et al.^94^ (2005)			
Artificial caries: 8 % methyl cellulose gel, 0.1 M lactate buffer, pH 4.6, 7 d and 28 d, for shallow (50 |jm) and deep (200 |jm) lesions, respectively	Bovine enamel (22mm2)	Ca loss and uptake (de-re solutions) , TMR (samples)	Dose-response was shown for Ca loss but not for Ca uptake. Significant difference was found for F response between shallow and deep lesions
pH cycling: (6x3 h/day):			
Re: (2.0 or 2.5 h and overnight, weekend, 1.5 mM CaCl_2_, 0.9 mM KH_2_PO_4_, 130 mM KCl, 20 mM Hepes, pH 7.0, 3 mL), rinse,			
De: (0.5 or 1h, 1.5 mM CaCl_2_, 0.9 mM KH_2_PO_4_, 50 mM acetic acid, pH 4.6-4.8, 3 ml) and rinse (1.5 mM CaCl_2_, 0.9 mM KH_2_PO_4_, 130 mM KCl, pH 7.0 unbuffered). The solutions were changed daily.			
pH cycling without treatment: 3 days			
Treatment: once/ day (moderate challenges) or twice/day (severe challenges) - 5 mL slurry (1:3 in water, 1x5 min or 2x/2 min)			
A robot was used for pH-cycling			
Ref: Ten Cate, et al.^88^ (2006)			
pH-cycling (7 d at 37°C):	Bovine enamel (4x4 mm)	S M H and CSH (samples) and Ca, P and F analysis (pH-cycling solutions)	The 550 ppm F acidified dentifrice had the same anticariogenic action as the 1,100 ppm F neutral formulation.
De: (2 mM Ca, 2 mM P, 0.04 ppm F, 75 mM acetate buffer, pH 4.7, 2.2 mL/mm^2^) for 6 h			
Re: (1.5 mM Ca, 0.9 mM P, 150 mM KCl, 0.05 ppm F, 0.1 M cacodylate buffer, pH 7.0, 1.1 mL/mm^2^) for 18 h			
Treatment: 1-min soak in slurries (1:3 water) between solution changes (twice a day). Last 2 days only in Re. According to Vieira, et al.^103^ (2005)			
Ref: Brighenti, et al.^11^ (2006)			
pH-cycling (7 d at 37°C):	Bovine enamel (4x4mm)	S M H, CSH, and analysis of F, Ca and P in enamel (microdrill biopsy technique)	The acidic dentifrices with 412 and 550 ppm F had the same efficacy as the neutral 1,100 ppm F dentifrice and commercial 1,100 ppm F dentifrice.
De: (2 mM Ca, 2 mM P, 0.04 ppm F, 75 mM acetate buffer, pH 4.7, 2.2 mL/mm^2^) for 6 h			
Re: (1.5 mM Ca, 0.9 mM P, 150 mM KCl, 0.05 ppm F, 0.1 M cacodylate buffer, pH 7.0, 1.1 mL/mm^2^) for 18 h			
Treatment: 1-min soak in slurries (1:3 water) between solution changes (twice a day). Last 2 days only in Re. According to Vieira et al.^103^ (2005)			
Ref: Alves, et al.^3^ (2007)			
Artificial caries (for re only): 0.05 M acetate buffer, pH 5.0 containing 1.28 mM Ca, 0.74 mM P, 0.03 ppm F, 2 mL/mm^2^for 32 h	Bovine enamel (4x4x3 mm)	SMH, CSH, PLM	The low-F dentifrice presented anticaries potential, but it was not equivalent to the dentifrices containing 1,100 ppm F
De pH-cycling (8 d, 37°C):			Both de and remineralizing models seem to be adequate to evaluate the anticaries potential of low-F dentifrice
De: 0.05 M acetate buffer, pH 5.0 containing 1.28 mM Ca, 0.74 mM P, 0.03 ppm F- 4 h/day, 6.25 mL/mm^2^			
Re: 1.5 mM Ca, 0.9 mM P, 150 mM KCl, 0.05 ppm F in 0.1 M Tris, pH 7.0 20 h/day, 3.12 mL/mm^2^			
Treatment: (before and after immersion in de): F solutions (0, 70, 140, 280 ppm F, NaF) or slurries (1:3) of dentifrices containing 0, 500 ppm F, 1,100 ppm F or Crest (1,100 ppm F - Gold standard) all NaF, for 5 min under agitation			
After the 8^th^cycle remained in Refor 24 h			
Re pH-cycling: same as De, but 2 h in De and 22 h in Re, 3 treatments of 1 min/day			
Ref: Queiroz, et al.^75^ (2008)			

**Figura 4:** pH-cycling studies comparing the efficacy of different fluoride compounds present in dentifrices for caries prevention

**PROTOCOL**	**DENTAL TISSUE**	**RESPONSE VARIABLE**	**CONCLUSION**
pH-cycling (6d + weekend only in Re solution, 37o C)	Human enamel	Reflected light microscopy, CSH, F/ Ca content by probe (samples) and F analysis in de-re solutions	NaF dentifrice and mouthrinse lead to a higher uptake of F into the lesion compared to MFP dentifrice, but the mineral content profile did not differ among the groups
De: 17h/day (40mL, 2 M Ca, 2mM PO4, 0.075M acetate, pH 4.3)	(4 windows 1x3mm, buccal and lingual)		
Re: 6h/day (20mL, 1.5mM Ca, 0.9mM PO4, 0.15mM KCl, 20mM cacodylate buffer, pH 7.0)			
Treatment :2x/day, dentifrice slurry 1:3 water or mouthrinse (1 min, under agitation)			
Ref: Nelson, et al.64 (1992)			
pH-cycling (7 or 14d at 21°C):	Human dentin	Fluoride analysis in samples (microdrill technique), TMR	The model provided reproducible results, demonstrated significant dose-related differences in the effects of both NaF and NaMFP-containing dentifrices on dentin F uptake and De, and detected a F-induced reduction in dentin caries, relative to a non-F control, similar to results established in a clinical trial
De: (0.1M lactic acid, 0.2% Carbopol 907, 50% saturated with hydroxyapatite, pH 5.0) for 4h/day [White110 (1987)]	(1mm in diameter window)		
Re: pooled human stimulated saliva for 20h			
Treatment: slurry (1:3 in water or human saliva) for 1min (4x1min/ day, 2 before and 2 after De)			
Initially pellicle formation for 0.5h in saliva			
Ref: Dunipace, et al.25 (1994)			
Artificial caries: demineralizing solution for 96h, 10mL/sample	Human enamel	TMR and PLM	Chinese and Indian dentifrices failed to show "healing" efficacy even though they claimed to contain varying levels of F.
pH cycling (10d):	(1mm window of molars)		
De: (2.2mM CaCl2, 2.2mM NaH2PO4, 0.05M acetic acid, pH 4.4 adjusted with 1M KOH, 2x3h/day)			
Re: (1.5mM CaCl2, 0.9mM NaH2PO4, 0.15M KCl, pH 7, 2h between DE/day, overnight). According to Ten Cate and Duijsters87 (1982)			
Treatment: slurry 1:3 in water 1min, 5mL/section, 3 times/day (before 1st De and before and after 2nd De).			
The solutions were replaced in each challenge			
Ref: Itthagarun, et al.43 (2000)			
Artificial caries: demineralizing solution for 96h, 10mL/sample	Primary enamel	TMR and PLM	Colgate Pokemon remineralized initial carious lesions, but Perioe children's toothpaste did not.
pH cycling (7d):	(1-mm window)		
De: (2.2mM CaCl2, 2.2mM NaH2PO4, 0.05M acetic acid, pH 4.4 adjusted with 1M KOH, 2x3h/day)			
Re: (1.5mM CaCl2, 0.9mM NaH2PO4, 0.15M KCl, pH 7, 2h between De/day, overnight).			
According to Ten Cate and Duijsters87 (1982)			
Treatment: slurry 1:3 in water 1min, 5mL/section, 3 times/day (before 1st De and before and after 2nd De).			
The solutions were replaced in each challenge			
Ref: Itthagarun, et al.42 (2007)			
Artificial caries: 0.1M lactic acid and 0.2% Carbopol C907, 50% saturated with hydroxyapatite, pH 5.0, according to White110 (1987)	Human enamel	SMH and F uptake (microdrill biopsy technique and F electrode)	AmF and MFP only dentifrices were less effective in enamel remineralization than NaF only and NaF+MFP formulations. The inclusion of human saliva as product diluents is a critically important aspect to consider for any *in vitro*study and most importantly when testing either AmF or MFP containing formulations.
pH-cycling (5d):	(3mm diameter)		
natural saliva (1min)/ slurry (1:3 natural saliva) (1min)/natural saliva (1h)/ slurry 1min/ natural saliva (1h)/De solution (3h)/saliva (1h)/slurry (1min)/saliva (1h)/ slurry (1min)/saliva overnight			
Ref: Casals, et al.14 (2007)			
pH cycling (14 days, 37oC):	Human enamel	CSH	AmF and NaF dentifrices had the same effect. However, NaMFP, without hydrolysis, had nearly no effect.
De: 6h/day (40mL, 2mM Ca, 2mM PO4,0.075M acetate, pH 4.3)	(4x2 mm)		
Treatment :(dentifrice slurry 1:3 in water and solution, 1min, under agitation)			
Re: 17h/day and on the weekend (20mL, 1.5mM Ca, 0.9mM PO4, 0.15mM KCl, 20mM cacodylate buffer, pH 7.0) according to Ten Cate and Duijsters87 (1982)			
Ref: Toda and Featherstone101 (2008)			

**Figura 5:** pH-cycling studies comparing the impact of new active principles on the anti-caries efficacy of fluoride dentifrices

**PROTOCOL**	**DENTAL TISSUE**	**RESPONSE VARIABLE**	**CONCLUSION**
pH-cycling (14 d, 37°C):	Human enamel	CSH	Inclusion of pyrophosphate in NaF dentifrice did not affect the net outcome of the cycling De/Re
De: 6 h/day (2 mM Ca, 2 mM PO_4_, 0.075 mM acetate, pH 4.3, 40 mL/sample)			
Treatment: 5 min dentifrice slurry 1:3 water (4 mL/sample, under agitation), water rinse			
Re: 17 h (1.5 mM Ca, 0.9 mM PO_4_, 150 mM KCl, cacodylate buffer pH 7, 20 mL/sample). Solutions were changed each 7d. On the weekends, there was only remineralization. According to Featherstone et al.^29^ (1986)			
Ref: Featherstone, et al.^30^ (1988)			
pH-cycling (6x3 h/day):	Bovine enamel	Ca uptake and loss (solutions)	The addition of triclosan and zinc citrate does not affect the caries-preventing property of F dentifrice
Re: (2.5 h and overnight, weekend, 1.5 mM CaCl_2_, 0.9 mM KH_2_PO_4_, 130 mM KCl, 20 mM cacodylate buffer, pH 7.0, 3 mL)			
De: (0.5 h, 1.5 mM CaCl_2_, 0.9 mM KH_2_PO_4_, 50 mM acetic acid, pH 5, 3 mL), according to Ten Cate and Duijsters^87^ (1982) with slight modifications.			
Treatment: 1 min daily in slurry (1:3 in water) followed by water rinse			
A pH-cycling robot was used to change the solutions			
pH cycling without treatment: 3 days			
pH cycling with treatment: 14 days			
Ref: Ten Cate^84^ (1993)			
pH-cycling: dentifrices were applied to sound enamel windows for 3 min at 8-h intervals for 14 d.	Human enamel	PLM (lesion depth)	The addition of ACaPO4 to a fluoride dentifrice resulted in a trend toward further reductions in lesion depth following *in vitro*lesion formation and progression over those obtained with a fluoride dentifrice.
Dentifrices were removed, enamel rinsed for 3 min with deionized water and placed in artificial saliva (20 mM NaHCO_3_, 3 mM NaH_2_PO_4_, 1 mM CaCl_2_, pH 7.0), rinsed with deinized water for 3 min.			
Artificial caries: enamel lesions were created with an acidified gel (1 mM Ca, 0.6 mM PO_4_, 0.1 mM F, pH 4.25) and evaluated by PLM.			
Treatment: enamel with caries-like lesions were treated again for 14 d as described above, returned to acidified gels for progression of the lesions and sections for PLM were obtained again. This was repeated once more.			
Ref: Hicks and Flaitz^39^ (2000)			
Artificial caries: 13 mL of 0.1 M lactic acid, 0.2% poliacrilic acid (Carbopol C907), 50% saturated hydroxyapatite, pH 5.0 for 72 h, according to White^110^ (1987).	Human enamel	SMH	The new dentifrice with ion-exchange resin (calcium, phosphate, fluoride and zinc) has the same effect than the conventional dentifrice in de/remineralisation
Treatment: 1 min, 10 mL slurry 1:3 in human saliva, 4 x/day	(0.6 cm diameter)		
Re: 15 mL natural saliva, 37°C, 1 h, under agitation			
De: 3 h in the same solution for producing artificial caries			
Total: 16 days (except weekends)			
Ref: Torrado, et al.^102^ (2004)			
Artificial caries: 0.2% carbopol C907, 0.1 M lactic acid 50% saturated with calcium phosphate, pH 5.0 for 44 h	Bovine enamel	S M H /p H of demineralizing solutions after the third dentifrice treatment	Dentifrice containing both F and sanguinaria was more effective than dentifrice containing F alone on remineralization of enamel lesion and on the pH of de solution. NaF dentifrices were more effective than MFP dentifrices.
Preparation: specimens placed in natural saliva for 24 h for pellicle formation/salivary mineral salts KCl, K_2_HPO_4_, NaCl, MgCl_2_and CaCl_2_were added to TSB containing 10% sucrose/Specimens were placed in 20 mL of TSB De-Re solution containing 2 mL of S. sobrinus (B13) cultured for 24 h/Culture for 24 h (twice)	(3 mm diameter)		
pH-cycling (15 days): 2 min in slurry (1:2 saliva), 2 h Re (50% stimulated human saliva and 50% artificial saliva), 2 h De (TSB with mineral salts and sucrose). This was repeated 3 times, but in the last time Re lasted 6 h and De 10 h			
Ref: Hong, et al.^40^ (2005)			
pH-cycling (14 days, 37ºC):	Human enamel (4x4 mm)	SMH	The whitening toothpastes evaluated showed effect similar to regular, nonwhitening toothpastes.
De: 6 h/day (24 ml, 2 mM Ca, 2 mM PO_4_, 0.075 M acetate, pH 4.3)			
Treatment: dentifrice slurry 1:3 in water, 5 ml 10 min			
Re: 17 h/day and overnight/ on the weekends (24 ml, 1.5 mM Ca, 0.9 mM PO_4_, 0.15 mM KCl, 20 mM HEPES buffer, pH 7.0) according to Featherstone et al.^29^ (1986)			
Ref: Watanabe, et al.^105^ (2005)			
			
Artificial caries: 2.2 mM CaCl_2_, 2.2 mM KH_2_PO_4_, 0.05 M acetic acid, pH 4.4, 96 h, 10 ml, 150-200 pm deep	Human enamel	PLM and TMR	Both test Asiatic dentifrices remineralized initial carious lesions. However, the remineralizing potential of Colgate Total was higher.
pH-cycling (10 d):			
Treatment : slurry (1:3), 5 ml, 1 min			
De: same as artificial caries, 10 ml, 3 h			
Re: 1.5 mM CaCl_2_, 0.9 mM NaH_2_PO_4_, 0.15 M KCl, pH 7.0, for 2 h			
Treatment: slurry (1:3), 5 ml, 1 min/demineralization solution 3 h/treatment with slurry (1:3), 5 ml 1 min/remineralizing solution overnight			
Ref: Rana, et al.^76^ (2007)			
Artificial caries (for Re only): 1:1 8% methyl cellulose/acid lactic gel system at 37°C, pH 4.6 for 10 days	Bovine enamel	Analysis of total Ca in acidic buffer with the electrode for De and % SMH change for Re	In de and remineralization studies, the silica based blue covarine whitening dentifrice was similar to the conventional dentifrice.
Re pH-cycling (6 x/day for 8 days. Neutral buffer overnight/ weekend)			
Treatment: slurry (1:3) for 5 min			
De: acidic buffer (1.5 mM CaCl_2_.2H_2_O, 0.9 mM KH_2_PO_4_, 130 mM KCl, 50 mM acetic acid, pH 5.0) for 30 min/neutral buffer (1.5 mM CaCl_2_.2H2O, 0.9 mM KH_2_PO_4_, 130 mM KCl, 20 mM HEPES ) for 10 min based on Gibbs et al.^34^ (1995)			
De pH-cycling (12 times, 2ml each solution): slurry (1:3) for 5 min/ acidic buffer (1.5 mM KH_2_PO_4_, 50 mM acetic acid, pH 5.0) for 60 min/neutral buffer (1.5 mM K H_2_PO_4_, 20 mM HEPES, pH 7.0) for 1 min based on Page^69^ (1991)			
Ref: Joiner, et al. ^45^ (2008)			

**Figura 6:** pH-cycling studies comparing the association between fluoride dentifrices and other treatments for caries prevention and treatment

**PROTOCOL**	**DENTAL TISSUE**	**RESPONSE VARIABLE**	**CONCLUSION**
pH-cycling (3d at 37°C):	Human dentin	SMH	The cariostatic effect shown by fluoride-containing dentifrice could enhance that shown by Ketac-FIl and Fuji II LC, and could mask that shown by F2000.
De: (1h) 2 mM Ca, 2 mM phosphate, acetate 74 mM, pH 4.3	(5x5x2mm)		
Re: (23 h) 1.5 mM Ca, 0.9 mM phosphate, 20 mM TRIS, pH 7.0			
Treatment: Slurry (1:3 in water) 2X5 min/day (after de and after re)			
According to Featherstone et al.^29^ (1986)			
Ref: Hara, et al.^36^ (2002)			
Artificial caries: According to White^108^ (1987) but 0.2% Carbopol 2050 was used	Bovine enamel	% SMH recovery and fluoride concentration in enamel (acid biopsy)	Although a single F-varnish application Is able to Increase fluoride concentration in enamel presenting early caries lesion, it does not improve the capacity of fluoride dentifrice used regularly in enhancing the enamel surface rehardening.
Re pH-cycling (12 d, 37°C): according to White^108^	(5x5 mm)		
De: 2 h/day, solution for lesion preparation, 0.037 ppmF			
Treatment: for dentifrices, 4 x/day, 50 ml slurry (1:3), 1 min; for varnish, single application, removal after 24 h			
Re: artificial saliva according to Ten Cate and Duijsters^87^ (1982), 0.049 ppm F, for the remaining of the period			
Ref: Maia, et al.^57^ (2003)			
pH-cycling (14 d with 10 cycles. In days 6, 7, 13 and 14 only Re ): According to Featherstone, et al.^29^ (1986)	Human enamel (4x4x3 mm)	Visual examination by 5 examiners scoring the presence and severity of caries-like lesions according to a scale ranked 0 to 3	The association of restorative materials and F dentifrice yielded higher cariostatic effect, except for the conventional glass ionomer cement, whose cariostatic effect was not influenced by the type of dentifrice
De: 2 mM Ca, 2 mM PO_4_, 0.075 M acetic acid, pH 4.3 15 ml for 6 h			
Treatment: dentifrice slurry (1:3) 5 ml for 5 min			
Re: (1.5 mM Ca, 0.9 mM PO_4_, 50 mM KCl, 20 mM Tris, pH 7.0) 15 ml for 18 h			
Ref: Rodrigues, et al.^77^ (2005)			
Artificial caries: 0.05M acetate buffer, 50% saturated with HAP, 48 h, 37ºC, 6.25 mL/mM2	Human enamel	F analysis (de-re solutions) and quantitative PLM and CSH (samples)	All treatment reduced the demineralization progress in enamel. However, the laser irradiation did not improve the effect of F.
Treatment: laser (once), F dentifrice slurry 1:3 water (2 x/day,5 min, under agitation, before De and Re), F mouthrinse (once, 1 min, under agitation, before De)	(4 mm^2^)		
pH-cycling: 10 d, 37ºC			
De: 5 mL/mm^2^, 2 mM Ca, 2 mM PO_4_, 75 mM acetate buffer, pH 4.6, 3 h/day			
Re: 2.5 mL/mm^2^, 1.5 mM Ca, 0.9 mM PO_4_, 150 mM KCl, 20 mM cacodylic buffer, pH 7.0, 21 h/day, after 5 d and at the end of experiment- 2 d			
Both solutions were changed daily			
Ref: Steiner-Oliveira, et al.81 (2008)			

It is required that the demineralizing procedures induce caries-like (subsurface lesion with a lessdemineralized surface layer) rather than erosionlike lesions. Many factors are important for the preservation of the surface layer, such as the presence of calcium and phosphate^19^ and fluoride^99^ in the solution, its pH^97^ and the time after an initial demineralization^98^, as the saturation might be reached with time, depending on the volume and viscosity of the demineralization solution relative to the area of enamel exposed to it. These factors interact with each other, making difficult the establishment of their ideal individual characteristics in a pH-cycling model. The formation of the surface layer might be also affected by agitation, whereas a higher agitation increases the risk of surface dissolution (Figure 7). It is important to consider these factors because the thickness of the surface layer may have influence on subsequent de-remineralization54.

Additionally, porosity and depth of a lesion can characteristics in a pH-cycling model. The formation also play an important role in mineral diffusion^54,88^. It of the surface layer might be also affected by has been recently shown that lesions with increasing ∆Z at baseline have a marked decrease in further mineral loss and a concomitant increase in further mineral gain. The decrease in demineralization of lesions with higher ∆Z at baseline may be partially a result of decreased intrinsic solubility through modified chemical composition such as loss of carbonate and magnesium. On the other hand, the tendency towards mineral gain might be due to the fact that larger, more porous lesions might be more easily remineralized than smaller and less porous lesions^83^. This may explain the tendency toward net remineralization with increasing ∆Z at baseline in pH-cycling regimes^55^. Furthermore, shallow lesions are more prone to demineralization than deeper ones because in deeper lesions the dissolved mineral from the deeper portions might undergo reprecipitation during outward diffusion^61,78^. On the other hand, the remineralization rate is lower in deeper lesions due to the longer distance for ionic diffusion before mineral deposition can occur^54,61^.

**Figure 8:**
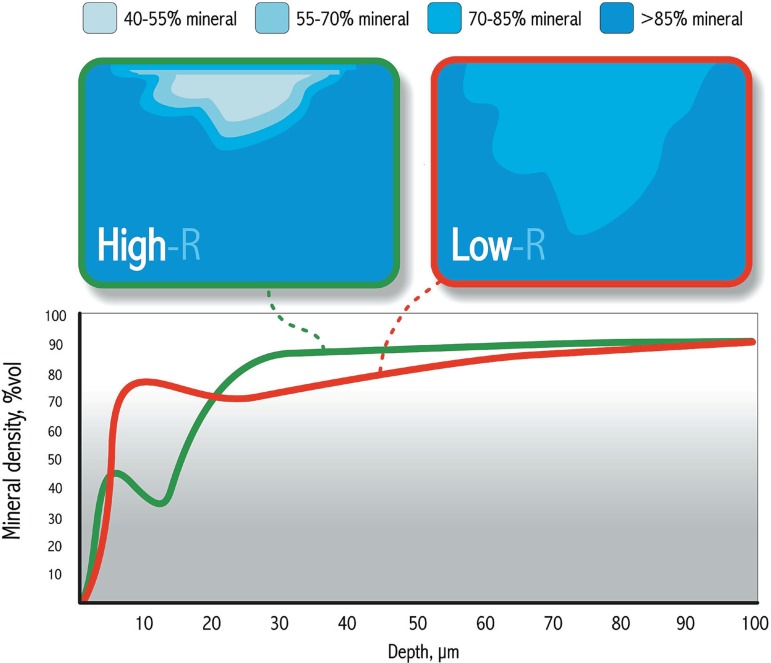
Mineral distribution in low-R and high-R caries lesions. High-R lesions give better discrimination among the treatments under study and seem to be more appropriate to compare the efficiency of remineralizing systems, such as fluoridated dentifrices. Low-R lesions are more appropriate when physiological mineral distribution is required. Modified from Lynch, et al.^54^ (2007)

In addition to the initial mineral loss of the artificial caries lesion, its mineral distribution is also very important. Low-R (R=∆Z/depth) lesions are more appropriate when physiological mineral distribution is required, while high-R lesions give better discrimination among the treatments under study and seem to be more appropriate to compare the efficiency of remineralizing systems^54^, such as fluoridated dentifrices (Figure 8). Thus, the comparison of the results from studies in which lesions with different characteristics at baseline were employed must be done with caution. Furthermore, fluoride dose-response studies should be carried out with lesions of various degrees of severity before conclusions on optimal fluoride efficacy are drawn^88^.

### Response Variables in ph-Cycling Models to Evaluate Fluoridated Dentifrices

Many response variables can be employed in pH cycling models to evaluate the efficacy of fluoride dentifrices. Some of them (colorimetric methods and atomic absorption spectrometry-AAS) evaluate the ions (mainly calcium and phosphate) released into the de- and remineralizing solutions. Also the calcium fluoride-like material and fluorapatite formed onto the dental substrates as a consequence of the treatments can be removed by alkali or acid biopsies and fluoride can be analyzed using an ionspecific electrode. From these biopsies, the amounts of calcium and/or phosphate can be also evaluated by the methodologies described above.

Regarding the analysis of the samples, depthrelated properties of artificial lesions can be described quantitatively by mineral content and hardness profiles^56^ and qualitatively by polarized light microscopy-PLM^39^ (more frequently) and scanning electron microscopy-SeM (less frequently)^114^.

Transverse microradiography (TMR) can be regarded as the "gold standard" for the evaluation of mineral distribution in Cariology research. This technique provides a quantitative measurement of the amount of mineral, lesion depth and surface layer thickness^31,48^.

More recently, microcomputed tomography (Micro-CT) has been used to study teeth. Carious lesions^23^, quantification of mineral distribution in fissure enamel^24^ and enamel de-and remineralization^21^ have already been studied by this method. It shows promise as a non-destructive method of validation for the presence and depth of tooth de-remineralization^21^. Precise measurements of attenuation coefficients and greater sensitivity to changes in mineral with time and position are provided by this technique. These benefits allow mineral content measurements from intact enamel to extensive demineralization, including a longitudinal tracking of lesion development. Besides, it allows complementary analyses of fluoride, calcium and phosphorus present in the enamel.

Hardness reflects the mechanical resilience of the substrate to the penetration of an indenter. Surface hardness employed with a reduced load (25-50 g) presents a good sensitivity to evaluate early changes (both de and remineralization) in the outermost layer of enamel and to predict the outcome of an anti-caries treatment^116^. It is important to highlight that when surface hardness is intended to be used as response variable in root caries models, the length of the pH-cycling must be shortened (from 10 to 3 days), as well as the length of the demineralization period (from 6 to 1 h) in order to enable the surface hardness of the created lesion to be assessed^36^. Cross-sectional hardness (CSH) can be regarded as an indirect method for evaluation of the mineral distribution because the technique, in fact, evaluates the resilience of the dental substrate to the penetration of an indenter under a given load. Despite different equations have been proposed and used to indirectly derive the mineral volume from the hardness data^31,48^, the correlation between hardness and mineral volume is different depending on the deepness of the lesion^21,56,87^. Additionally, the size of the indentation in demineralized tissues only allows the measurement of cross-sectional hardness at distances of at least 20 µm between two indentations, which might result in inaccuracy when the integrated mineral loss is calculated^21^.

Another limitation of hardness measurement is that the size of the indentation highly depends on the organic and water contents of the tissue. This fact has a especial impact on dentin that seems to present high variation of hardness values depending on the degree of hydration, which, in turn, might compromise the analytical results^7^.

Recently, different methodologies to produce artificial carious enamel lesions (2 gels, 2 buffered solutions and a pH-cycling protocol) were tested using TMR and cross-sectional hardness as response variables. One interesting result of this study was that both formulas to convert hardness to mineral volume^31,48^ presented overall high correlation between them and low correlation with TMR data, showing that the conversion of the crosssectional hardness to mineral volume seems to be inadequate. Thus, the data should be expressed as hardness numbers. The results also indicate that the conversion of hardness to mineral volume should not be used, since the formulas and the TMR data showed different results when they were used to compare the five methodologies at each depth. This finding gives more support to the fact that the results are dependent on the protocol used for creating artificial lesions^56^.

### ADA Profile Applications of Dentifrices in ph-Cycling Models

Regardless the type of model employed, it must meet the suggested ADA guidelines associated with topical evaluation of dentifrices and determination of efficacy in products^1,2,17,73,74^. The model must preserve two important properties that relate it to the caries process and to the investigator performing the experiment: validity and reliability. The validity of a model can be defined as the degree of success with which the model actually provides information about the phenomenon or process it is being used to study, i.e., the pH-cycling protocol should be able to simulate as closely as possible the clinical situation.

Reliability is related to the manner by which an investigator obtains measurements in an experiment. The reliability of a measurement technique assesses the degree to which similar values would tend to be obtained if repeated measurements were made on the same test sample, under nearly identical conditions^73^. Furthermore, a pH-cycling model must show dose-response effect differentiating dentifrices containing 0, 250 and 1,000 or 1,100 ppm F. It is not mandatory that the models are able to differentiate the anti-caries effect of a low-F dentifrice containing 500-550 ppm F from the conventional 1,000-1,100 ppm F. However, it is recommended to include a 500 ppm F treatment group to obtain a "mini dose-response"^74^.

### Controls

A working group report on laboratory models for caries^91^ recommended that for tests of fluoridated dentifrices in the traditional concentration range (0-1,100 ppm F), experimental groups treated with controls of 0, 250 and 1,000 or 1,100 ppm F (a clinically proven "gold standard") are required, since clinical data are available on fluoride efficacy in this concentration range. The purpose of the 250 ppm F dentifrice would be to serve as an intermediate "dose-response" control that should generally produce results that are 10 to 20% better than the placebo, but not as effective as the 1,000 ppm F "gold standard". The 250 ppm F dentifrice for tests can be made especially by the manufacturer or by dilution of the 1,000 ppm F dentifrice, and this should then be tested for bioavailability. For studies with products in other concentration ranges, other controls should be included, including the concentration range tested. Treatment should be done with slurries of 1 in 3 (or 1 in 4) weight/weight dilutions of the toothpastes in water or saliva^91^.

### Statistical Considerations

A working group report on laboratory models for caries^91^ agreed that the PCK (Proskin-ChiltonKingman)^74^ test, originally designed for intra-oral studies^73^, may also be an appropriate statistical test for *in vitro* pH-cycling models to evaluate products for "equivalence". By definition, a test product is considered equivalent to a control product if its effect is between 90% and 110% of that of the control; that is, if the ratio of true mean effectiveness scores (test over control) is between 0.9 and 1.1. This range of values is the "range of equivalence"^73^.

To establish the equivalence of a test product to a control, the guidelines specify two methods. The first involves either a confidence interval or, alternatively, the use of two one-sided hypothesis tests. The second method is the "power rule". Details on how to conduct these tests were described by Proskin^73^ (1992).

### 3.5 ph-Cycling Protocols to Evaluate the Anti-Caries Efficacy of Fluoride Dentifrices

Since the classical study by ten Cate and Duijster^87^ (1982), who used a pH-cycling model to show that fluoride (100 µmol/L) present during the remineralization phases causes lesion arrestment, several pH-cycling protocols have been proposed and employed to evaluate the efficacy of fluoride dentifrices. These are summarized in Figures 3, 4, 5 and 6 according to the focus of the studies: dose-response or pH-response of fluoridated dentifrices, comparison of different F compounds in dentifrices, the impact of new active principles on the effect of fluoridated dentifrices and the effect of fluoridated dentifrices associated to other treatments, respectively. As it can be seen, the composition and volume of the de-remineralizing solutions tested varies substantially in the different protocols. Some studies show the volume of the solution by area of the sample. This is important information that should be standardized in all papers. Also the length of the treatments spans a wide range (mainly 7-15 days) and many response variables can be used to assess the result of the treatments (mainly chemical analysis of solutions and physical analysis of the samples).

One of the most often used pH-cycling protocols is the one described by Featherstone, et al.^29^ (1986) for human enamel, which was modified from the one proposed by ten Cate and Duijsters^87^ (1982). The model is of particular interest because it simulates *in vivo* high caries risk condition (lesions formed around orthodontic banding following 1 month *in vivo*)^67^ and simultaneously measures the net result of the inhibition of demineralization and the enhancement of remineralization. In this model, the dynamic cycles of de- and remineralization are simulated by sequentially immersing enamel specimens in acidic (demineralizing) and supersaturated (remineralizing) buffer solutions. Dentifrice use is simulated by immersing specimens in slurries during the de- and remineralization stages. Topical efficacy is subsequently evaluated in terms of the ability of a test product to limit caries progression as a result of enhanced remineralization and/or diminished demineralization. The demineralization stage (6 h) uses an acid buffer containing 2 mM Ca (Ca(NO_3_)_2_), 2 mM PO_4_(KH_2_PO_4_) and 75 mM acetate at pH 4.3. The remineralization solution (17 h) contains calcium and phosphate at a known degree of saturation (1.5 mM Ca and 0.9 mM PO_4_) to mimic the remineralizing properties of saliva, 130-150 mM KCl (to provide background ionic strength) and 100 mM TRIS or 20 mM cacodylate buffer at pH 7.0. This solution approximates the mineral ion composition and supersaturation of saliva as originally reported by ten Cate and Duijsters^87^ (1982). Some authors (Figures 3-6) changed the exposure time in de- (3 h, 6 h or 17 h/day) and remineralizing (6 h or 17 h/day) solutions or the concentrations of the ions, according to the focus of the study (de or remineralization). When the focus is more on remineralization, the samples stay less time in the demineralizing solution, and this solution is prepared with lower concentration of acid and ions and a higher pH. On the other hand, the time in the remineralizing solution is higher, but the concentration of ions and the pH remain unchanged (Figures 3-6).

Another common pH-cycling model for bovine enamel is recommended by ten Cate^84^ (1993). In this case, the demineralizing solution contains 1.5 mM Ca (CaCl_2_), 0.9 mM PO_4 _(KH_2_PO_4_), 0.050 M acetate at pH range of 4.6-5.0. The remineralization solution contains calcium and phosphate at a known degree of saturation (1.5 mM Ca and 0.9 mM PO_4_) to mimic the remineralizing properties of saliva, 130 mM KCl (to provide background ionic strength) and 20 mM HePeS or cacodylate buffer at pH 7.0. The cycles, in this case, are more frequent (6x 0.5 h in De/day and 6x 2.5 h in Re/day). Additionally, the addition of fluoride (0.03-0.06 ppm) into both de- and remineralizing solutions^3,11,75,86,103^ has been proposed. For primary teeth, it is recommended to add higher fluoride concentrations in the de- and remineralizing solutions (0.25 ppm F)^94^.

Usually, the samples stay in remineralizing solutions not only between the demineralizing challenges, but also overnight, on the weekends and sometimes all day in the last days. In some protocols the de-remineralizing solutions are changed daily, while in others they are not. This change can be made manually or by custom-made robots^84,88^. Another important point it is that in some papers, the pH cycles are performed for 3 days without the F dentifrice (slurry) treatment^86^ to allow baseline values of calcium uptake and loss to be determined. Then, the treatment is performed either before or after the demineralization challenge^93^, 1 to 3 times per day, for 1 to 5 min, under agitation, during 7 to 14 days.

While pH-cycling models using lactic or acetic acids are the most common approaches for testing fluoride dentifrices, the use of biotic models has been proposed, since microbial activity-dependent pH-cycling has been shown to be of benefit in testing dentifrices containing NaF and plant extracts, such as sanguinaria, considering that these plant extracts could affect the activity of microorganisms more than does NaF^40^. However, these models are of much more complex execution, which restricts their use.

pH-cycling models have been shown to be appropriate to show dose-response of NaF dentifrices (Figure 3), both in primary and permanent enamel ^3,11,69,75,86,93-95,101,107^ and permanent dentin^25,86,93^. In a clinical study, dentifrice usage was found to be effective both in preventing enamel and root dentin caries, with 41% and 67% reductions, respectively, in one-year caries increment data^44^. This is consistent with data from a pH-cycling study showing that fluoride given by means of a dentifrice treatment has a significantly more pronounced effect on dentin than on enamel^86^. Additionally, in the pH-cycling model proposed by Dunipace, et al.^25^ (1994) dentin demineralization was reduced by 65% when a 1,100 ppm F (NaF) dentifrice slurry was used, which is almost identical to the result obtained in the clinical trial^44^. However, constantly present low fluoride levels (3 µM in de- and remineralizing solutions) are inadequate to shift the de-remineralization balance sufficiently for dentin, as occurs with enamel. More fluoride is needed for remineralization of dentin than for enamel^38^. It has been speculated that the high concentration fluoride "pulses" that occur after treatment with dentifrices might facilitate secondary nucleation, increasing the internal surface area of dentin for crystallization.

While clinical trials show a beneficial effect of 5,000 ppm F over 1,100 ppm F dentifrices to arrest root carious lesions^9,53^, this was not tested in pHcycling studies. Such studies could help to clarify the mechanism of action of high-fluoride dentifrices on root caries arrestment. Additionally, the effect of fluoride is most pronounced during demineralization than during remineralization for both enamel and dentin^86^.

Also appropriate conclusions about pH-response of NaF dentifrices^3,11^ can be drawn from pHcycling studies (Figure 3). Low-fluoride (500 ppm) acidic (pH 4.5 or 5.5) NaF dentifrices have been shown in pH-cycling studies to be as effective as 1,100 ppm F neutral NaF dentifrices. This was confirmed in clinical studies showing that a 550 ppm F pH 4.5 dentifrice led to similar plaque fluoride concentrations as the 1,100 ppm F pH 7.0 dentifrice^12^ and that both dentifrices led to similar caries increments in a 20-month clinical trial with 4-5-year-old children (unpublished date).

One aspect that should be considered in studies involving testing of MFP or amine fluoride (AmF) based dentifrice formulations is the solution used to prepare the dentifrice slurry (Table 2). In these cases, it is important to use natural saliva to make the dilution^14^. For MFP-based formulations, this approach is necessary because salivary enzymes assist in the hydrolysis of the covalently bound MFP to release fluoride ions. It is important that the model is conducted at 37°C because it has been shown that when NaMFP slurries were prepared with fresh human saliva at 21°C, only minimal amounts of the total fluoride (less than 10%) were available as ionic fluoride^25^. Since the enzymatic system is efficient in the *in vivo* condition, no differences in the anti-caries efficacy between dentifrices formulated with NaF or MFP occur in the clinical situation, as revealed by recent meta-analyses^16,59^. The variable simulation of conditions necessary for MFP hydrolytic activity probably explains the large range of effects seen in *in vitro* comparisons of

NaF and MFP^6,20,25,35,40,42,43,49,62,85,101,107-109,111-113.^ It has recently been shown that the minor inhibitory effect of a NaMFP formulation (1,000 ppm F) on lesion formation of dental enamel was a result of the low concentration of free fluoride ion (only 30 ppm)^101^. AmF, in particular, has a very low pH when diluted with water (4.96) rather than saliva (6.37)^14^. When *in vitro* studies use water instead of saliva as the product diluents, results for AmF are generally more favorable^65,101^. This effect is generally considered to be an artifact of study design rather than product effectiveness when compared to protocols that use natural saliva for this purpose^14^. In the last decades there has been a proliferation of dentifrices directed toward specific needs of the population^13^. Such designer dentifrices include those directed against tartar, gingivitis, enamel and root caries, root sensitivity, halitosis, and tobacco stains, as well as those with extra-whitening abilities, and those considered to be "natural" dentifrices and dentifrices for children. Despite the various types of toothpastes, fluoride has been maintained in over 95 percent of the dentifrice formulations^39^. pH-cycling studies have been employed to evaluate if the addition of different active principles would interfere with the anti-caries action of fluoride (Figure 5). Overall, the addition of active principles with different purposes such as pyrophosphate (anticalculus)^30,71^, blue covarine^45^, carbamide peroxide^105^ and sodium hexametaphosphate^72^ (whitening), sanguinaria (antiplaque)^40^, ionexchange resins^102^, triclosan (antibacterial) combined with PVM/MA^39,76^ or zinc citrate^84^ have not jeopardized the caries-preventive properties of the fluoridated dentifrices in pH-cycling studies. Variations in model sensitivity can be observed in *in vitro* assessments of the activity of anticalculus dentifrices^107^, where some protocols show significant inhibition of remineralization with the addition of tartar control inhibitors to fluoride dentifrices, while others show no negative effects with the addition of these inhibitors^30,89,93,107,115^. However, clinical^51,52,82^ and *in situ*^63^ studies with tartar control dentifrices confirm that mineralization inhibitors do not appear to affect caries protection of dentifrices, in-line with data reported from some pH-cycling studies. For whitening dentifrices, there is only scanty information on their anti-caries efficacy and there is still a need for further evaluation of both different types of compounds and lesions in pH-cycling and *in situ* studies. On the other hand, the addition of bioavailable amorphous calcium and phosphate in a NaF dentifrice seems to cause a trend toward further reduction in lesion depth over fluoridated dentifrice in a pH-cycling model simulating caries formation and progression^39^.

pH-cycling models have also been employed to verify the performance of the association between fluoridated dentifrices and other measures for caries prevention such as CO_2 _laser irradiation^81^, F varnishes^57^, F solutions^64^ or caries treatment such as fluoride-releasing or not dental materials^22,36,77^ (Figure 6). The association between fluoridated dentifrices and other caries-preventive measures, such as CO_2_ laser irradiation^81^ or fluoride varnishes was not able to increase the cariostatic action of the F dentifrices in pH-cycling protocols. This is in agreement with a recent meta-analysis showing that the combination of fluoride dentifrice with other topical fluoride treatments (mouthrinses, gels, varnishes) for preventing dental caries in children and adolescents had only a mild additive effect (around 10%) over dentifrice alone^58^, indicating that pH-cycling models might be appropriated for this kind of evaluation.

More recently, attention has been devoted to the development of pH-cycling models that would be appropriate to test the efficacy of fluoridated dentifrices on de/remineralization of primary teeth. It has been investigated if the *in vitro* 10-day pHcycling model used for permanent teeth^43,87^ could be used to evaluate de/remineralization effects of child formula toothpastes on the enamel of primary teeth^95^. It was observed that by day 8 all lesions in the sections were eroded and/or had progressed into dentin. This may have been due to the fact that the enamel of primary teeth has lower enamel thickness and mineral content and higher organic content when compared with permanent teeth. Due to the rapid formation of enamel in primary teeth, it may have more imperfections in the hydroxyapatite crystals than permanent enamel. These variations in structure are known to influence caries susceptibility, acid etching characteristics^114^ and give lower initial hardness values for primary teeth^8^. Thus, if this pH-cycling model is intended to be used for primary teeth, a reduction from 10 to 7 days in length is recommended. However, shortening of the period of pH-cycling might produce results that inadequately represent the natural process of de- and remineralization. The factors that influence the length of the pH-cycling are the pH and the fluoride concentration of the de- and remineralizing solutions^34^. The de- and remineralizing solutions used in the study by Thaveesangpanich, et al.^95^ (2005) did not contain additional fluoride. In the absence of fluoride, even under conditions that are considered to favor remineralization, further demineralization has been shown to take place^34^. Thus, in a subsequent study, it was tested if the addition of 0.25 ppm F in the de- and remineralizing solutions of the 10-day pH cycling model would lead to similar rates of lesion progression when compared to 7-day pH-cycling models where no fluoride was added to de- and remineralizing solutions. The authors observed that the two models led to similar results regarding lesion progression. Thus, a 10-day pH-cycling model similar to that used for permanent teeth^43^ can be used for primary teeth provided the remineralizing and demineralizing solutions contain 0.25 ppm F^94^.

### Future Perspectives in ph-Cycling Protocols

Research should be focused to fill the gap between *in vitro* models and the *in vivo* situation. Thus, *in vitro* models should be improved regarding their predictive value. Currently, there is a limited number of studies that use solutions that simulate plaque fluid conditions^27,54^ and future studies should focus on this. It would be interesting to design models where the solutions mimic plaque fluid ionic concentration and pH in individuals with low and high caries risk, after fasting and exposure to sucrose, in order to model various caries risk situations. Additionally, the models could include the addition of organic salivary components, a simulation of dental plaque, temperature, volume effects and brushing conditions. Ideally, all these conditions should be included in automated models.

Regarding root caries, it is important to develop models that more closely resemble the clinical progression and reversal of caries in dentin, taking into account not only the mineral content, but also its organic matrix. These models could be used to study the influence of the addition of MMPs inhibitors^33,46^ or collagen cross-linkers^104^ to fluoridated dentifrices on their anti-caries potential in dentin.

It would also be very useful trying to validate pH-cycling models against *in situ* challenges with sucrose in the absence or presence of fluoride in different concentrations. If comparable results are obtained, then pH-cycling models can be used instead of *in situ* protocols that are more timedemanding and difficult to be conducted.

## CONCLUSIONS

Critical features of pH-cycling models to evaluate the efficacy of fluoridated dentifrices on caries control include the ability to detect known results established by clinical trials (ADA specifications), to demonstrate dose-related responses in the fluoride content of the dentifrices, and to provide repeatability and reproducibility between tests. In order to accomplish these features satisfactorily, it is mandatory to take into account the type of substrate and baseline artificial lesion, as well as the adequate response variables and statistical approaches to be used. If these aspects are adequately contemplated, the currently available pH-cycling models are appropriate to detect dose response and pH-response of fluoride dentifrices,to evaluate the impact of new active principles on the effect of fluoridated dentifrices, as well as their association with other anti-caries treatments. However, further studies should be done in order to make pH-cycling models as close as possible to *in vivo* conditions.
